# In Vitro Fertilization Using Preimplantation Genetic Testing in a Romanian Couple Carrier of Mutations in the TTN Gene: A Case Report and Literature Review

**DOI:** 10.3390/diagnostics11122328

**Published:** 2021-12-10

**Authors:** Bogdan Doroftei, Radu Maftei, Ovidiu-Dumitru Ilie, Theodora Armeanu, Maria Puiu, Iuliu Ivanov, Loredana Nemtanu

**Affiliations:** 1 Department of Mother and Child Medicine, Faculty of Medicine, University of Medicine and Pharmacy “Grigore T. Popa”, University Street, No. 16, 700115 Iasi, Romania; bogdandoroftei@gmail.com (B.D.); dr.radu.maftei@gmail.com (R.M.); 2 Clinical Hospital of Obstetrics and Gynecology “Cuza Voda”, Cuza Voda Street, No. 34, 700038 Iasi, Romania; 3 Origyn Fertility Center, Palace Street, No. 3C, 700032 Iasi, Romania; nemtanuvalentinaloredana@gmail.com; 4 Department of Biology, Faculty of Biology, “Alexandru Ioan Cuza” University, Carol I Avenue, No. 20A, 700505 Iasi, Romania; 5 Department of Microscopic Morphology, Faculty of Medicine, University of Medicine and Pharmacy “Victor Babeș”, Eftimie Murgu Square, No. 2, 300041 Timișoara, Romania; maria.puiu@gmail.com; 6 Molecular Diagnostic Department, Regional Oncology Institute, General Henri Mathias Berthelot Street, No. 2–4, 700483 Iasi, Romania; iuliuic@gmail.com

**Keywords:** severe congenital myopathy with fatal cardiomyopathy, titin, in vitro fertilization, preimplantation genetic testing, sanger sequencing, next-generation sequencing

## Abstract

Severe congenital myopathy with fatal cardiomyopathy (EOMFC) is a rare genetic neuromuscular disorder inherited in an autosomal recessive manner. Here we presented a successful pregnancy obtained by in vitro fertilization (IVF) using preimplantation genetic testing (PGT) in one young Romanian carrier couple that already lost mutation(s) within the TNN gene and whose first baby passed away due to multiple complications. It was delivered via emergency C-section at 36 weeks and fully dependent on artificial ventilation for a couple of months, weighing 2200 g and an APGAR score of 3. The aCGH + SNP analysis revealed an abnormal profile of the first newborn; three areas associated with loss of heterozygosity on chromosome 1 (q25.1–q25.3) of 6115 kb, 5 (p15.2–p15.1) of 2589 kb and 8 (q11.21–q11.23) of 4830 kb, a duplication of 1104 kb on chromosome 10 in the position q11.22, and duplication of 1193 kb on chromosome 16 in the position p11.2p11.1. Subsequently, we proceeded to test the parents and showed that both parents are carriers; confirmed by Sanger and NGS sequencing—father—on Chr2(GRCh37):g.179396832_179396833del—TTN variant c.104509_104510del p.(Leu34837Glufs*12)—exon 358 and mother—on Chr2(GRCh37):g.179479653G>C—TTN variant c.48681C>G p.(Tyr16227*)—exon 260. Their first child died shortly after birth due to multiple organ failures, possessing both parent’s mutations; weighing 2200 g at birth and received an APGAR score of 3 following premature delivery via emergency C-section at 36 weeks. Two embryos were obtained following the IVF protocol; one possessed the mother’s mutation, and the other had no mutations and was normal (WT). In contrast with the first birth, the second one was uneventful. A healthy female baby weighing 2990 g was delivered by C-section at 38 weeks, receiving an APGAR score of 9.

## 1. Introduction

Titin (TTN) (previously formerly known as named “connectin”) [1] is a very large sarcomeric protein of 3700 kD, mainly located in the skeletal muscles [2]. The entire coding region of the TTN consists of 363 exons, which encode 38,130 amino-acid residues of 4200 kD, leading to an overall physical length of 2 μm [3].

It is worth mentioning noting that TTN has indirect interactions with myotilin, with several ligand-binding sites for calpain-3, telethonin, and α-actinin. Mutations that may occur at the level of affecting these particular binding sites have been linked to various forms of other life-threatening dystrophies [4,5].

Heterozygous dominant mutations in the N-terminal, proximal, or distal distant regions of the TTN gene have been attributed to cardiomyopathy [6,7], hereditary myopathy with early respiratory failure (HMERF) [8,9,10], Emery–Dreifuss-like phenotype without cardiomyopathy (EDMD) [11], and severe congenital myopathy with fatal cardiomyopathy (EOMFC) [12].

The positive correlations found between the severity of cardiac affection and the location of the respective mutations indicate that: the closer the mutation is to the C-terminus, the less severe is the pathological panel manifestations. It should also be noted that homozygous recessive mutations within the N-terminal domain are lethal [12].

EOMFC, Salih myopathy (SAMLY), or Salih’s congenital muscular dystrophy (SALIH MYOPATHY, OMIM#611705) is a rare genetic neuromuscular disorder inherited in an autosomal recessive manner. It is mainly characterized by skeletal weakness and delayed motor development [13,14,15,16].

Unfortunately, the actual prevalence remains unknown since scare data has been found in the current literature and cases described by it have been described on only three distinct occasions by Carmignac [12], Chauveau [17], Salih [18] and co-authors. Furthermore, its pathophysiology is similar to that observed in patients diagnosed with limb-girdle muscular dystrophy type 2J (LGMD2J), which is allelic to EOMFC. The only difference is represented by the onset (neonatal period or in early infancy), both being caused by a mutation in the C-terminal region of the TTN gene [4,5].

Based on the aforementioned, here we present the case of a young Romanian carrier couple, both carriers of a mutation in the TTN gene mutations that obtained a second pregnancy through in vitro fertilization (IVF) using preimplantation genetic testing (PGT).

## 2. Case Presentation Section

### 2.1. First Unsuccessful Pregnancy

The mother, 34-years old, primigravida (G0P0), underwent all recommended tests. The first-trimester morphology scan revealed normal crown-rump length, visible nasal bone, and normal nuchal translucency value. Moreover, the double marker for chromosomal aneuploidies (13, 18, and 21) indicated a low-level risk. The TORCH IgM and IgG screening showed no acute or recent infection (negative IgM), and the IgG titer was high. The woman had not been previously exposed to harmful factors that would have justified placing the pregnancy in the high-risk category. The second-trimester morphology scan performed at 22 weeks confirmed the normal development of a female fetus.

However, at 33 weeks of pregnancy, the first abnormal sign was noted. The amniotic fluid quantity started to increase, leading to the diagnosis of polyhydramnios. Another visible alteration was the shape and position of the lower fetal limbs, indicating minor clubfoot and altered fetal biophysical profile. By the time the pregnancy reached 36 weeks, the biophysical variables were severely modified. The fetal heart rate monitored using the non-stress test was worrying. There were significant decelerations, abnormal fetal movement, and poor muscular tonus. Additionally, the quantity of amniotic fluid continued to rise. Cumulatively, these observations led to the decision to deliver the baby prematurely via emergency C-section, 36 weeks into the pregnancy.

The C-section was uneventful, and the mother made a fast recovery, but the female newborn weighing 2200 g received an APGAR score of 3. Unfortunately, when thoroughly examined by our team, it was noticeable that the fetus’s movement, breathing, and swallowing capacity were impaired, and she was unable to sustain spontaneous breathing. The newborn was constantly and fully dependent on assisted mechanical ventilation. Her condition continued to deteriorate despite all the efforts. Unfortunately, at two months of age, the baby succumbed to respiratory failure and multiple associated complications.

### 2.2. Initial Diagnoses of the Newborn

Based on the clinical signs and paraclinical tests, we were able to establish the following diagnostics: generalized congenital muscular atony, right diaphragmatic hernia, cerebral atrophy, neonatal anemia, bilateral varus equinus, neonatal hypocalcemia, prematurity and low birth weight, ostium secundum atrial heart defect, and tricuspid valve dysplasia.

Thoracic X-rays show reduced ribcage expansion of the right hemithorax, suggestive of right diaphragmatic hernia. The transfontanelar ultrasound and head CT showed moderate cerebral atrophy, mostly in the frontal lobe. The generalized and severe muscular hypotonia was investigated using a muscular biopsy that showed no significant alterations. Both tests for aminoacidopathies and spinal muscular atrophy were negative. Serum levels of creatine kinase (CK) and lactate dehydrogenase (LDH) were high but with a tendency to normalize. The karyotype showed a normal profile 46XX.

Given the multitude of heterogenic clinical symptoms, we suspected a genetic syndrome yet to be diagnosed, so we proceeded to perform an Array Comparative Genome Hybridization (aCGH) with Single Nucleotide Polymorphism (SNP). aCGH+SNP was conducted on a blade with 4 ∗ 180,000 (180 K) samples (110.112 CGH samples, 59.647 SNP samples, 3000 replicated samples and 8121 control samples) covering the entire human genome with a spatial resolution of ~25.3 kb DNA (G4890A, design ID: 029830, UCSC hg19, Agilent). The scans were interpreted with the CytoGenomics Agilent software, using standard interpretation parameters with a SureScan Microarray Scanner. The resulting profile was abnormal: there were three areas associated with loss of heterozygosity on chromosomes 1 (q25.1–q25.3) of 6115 kb, 5 (p15.2–p15.1) of 2589 kb and 8 (q11.21–q11.23) of 4830 kb, a duplication of 1104 kb on chromosome 10 in the position q11.22, and duplication of 1193 kb on chromosome 16 in the position p11.2p11.1.

### 2.3. Parents’ Testing

Considering this abnormal genetic profile, the parental couple received genetic counseling. Furthermore, we continued to test both partners through Next Generation Sequencing (NGS) by Illumina.

**Figure 1:**
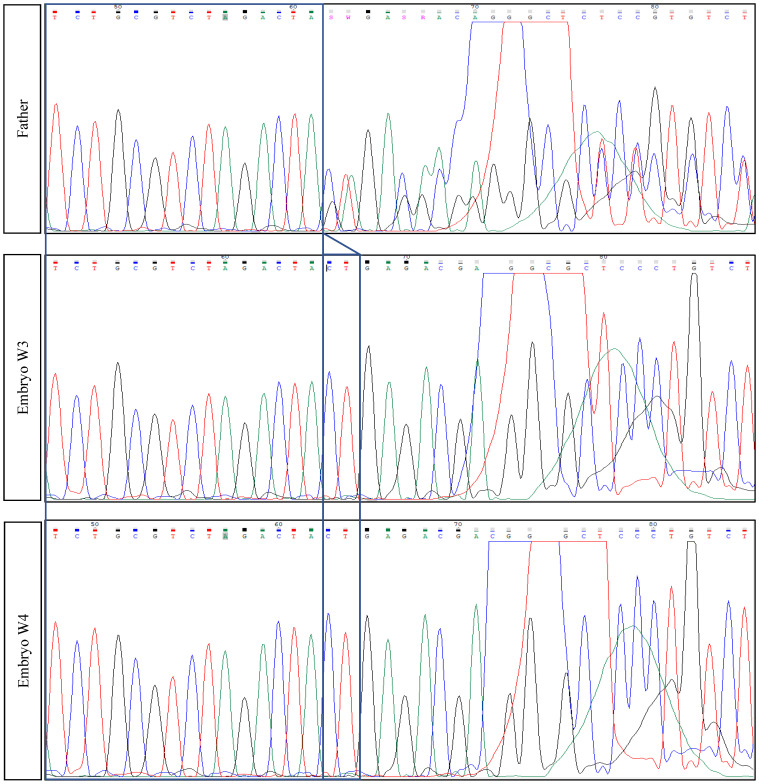

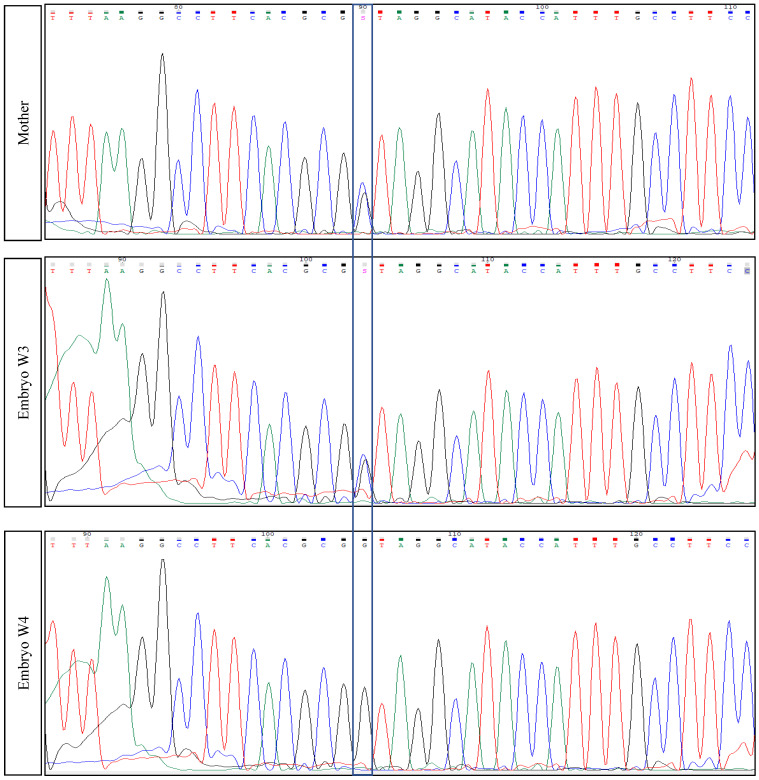
Electropherograms following Sanger sequencing in both parents and offspring; embryo(s) W3 and W4 did not possess the father’s mutation. W3 embryo possesses the mother’s mutation in contrast with the W4 embryo that was transferred.

The results confirmed the following abnormal genetic profile; TTN (NM_001267550.1, sequencing): heterozygous variant on Chr2(GRCh37):g.179479653G>C—TTN variant c.48681C>G p.(Tyr16227*)—exon 260, heterozygous variant on Chr2(GRCh37):g.179396832_179396833del—TTN variant c.104509_104510del p.(Leu34837Glufs*12)—exon 358 (TTN: NM_001267550.1—reference sequence). The TTN variant c.48681C>G p.(Tyr16227*) creates a premature stop codon. Sanger sequencing also confirmed this variant and was classified as likely pathogenic (class 2). The TTN variant c.104509_104510del p.(Leu34837Glufs*12) creates a shift in the reading frame starting at codon 34837. The new reading frame ends in a stop codon 11 positions downstream. This variant has been confirmed by Sanger sequencing, and it is also classified as likely pathogenic (class 2) (Figure 1).

### 2.4. Successful Pregnancy

**Figure 2:**
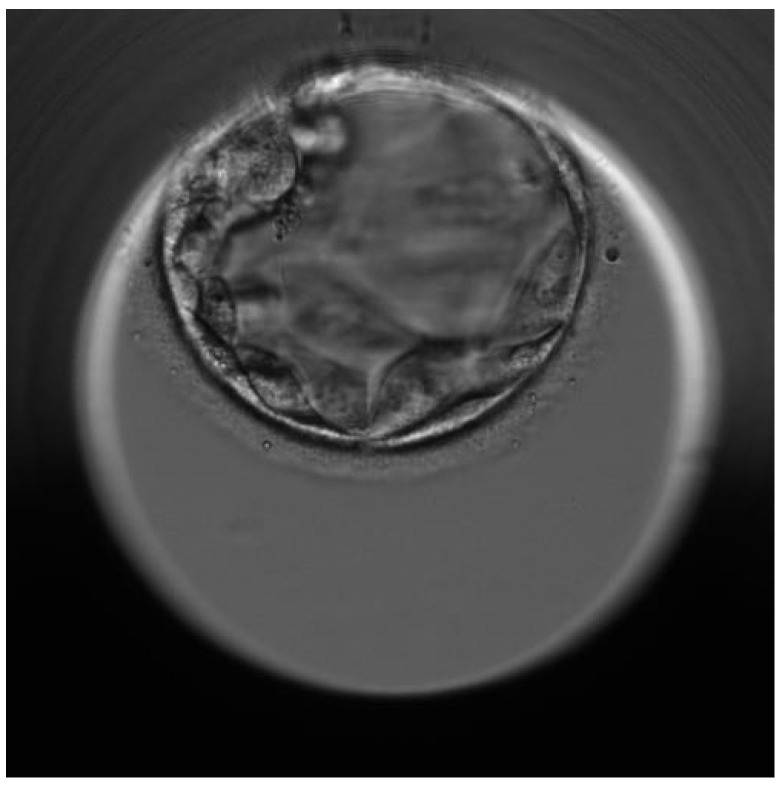
Embryoscope picture of the tested euploid day-5 embryo (blastocyst) that was transferred in a frozen-thawed cycle with endometrial preparation.

In light of the clinical outcomes, based on the previous unfortunate experience, the couple agreed to receive genetic counseling. The couple was advised to pursue in vitro fertilization (IVF) with preimplantation genetic testing (PGT-M). Considering the mother’s age and weight, her Anti-Müllerian hormone (AMH) serum level and antral follicle count, we used a short-antagonist ovarian stimulation (OS) protocol with 200 UI of FSH (follitropin beta) concomitantly with 150 UI of combined FSH and LH (menotropin). Ten days later, seven oocytes were retrieved through transvaginal, sonographically controlled follicle puncture. Five of them were injected through intracytoplasmic intracytoplasmatic sperm injection (ICSI), resulting in two blastocysts. The embryonic biopsy was performed on day 6 of the blastocyst stage for these two embryos (Figure 2).

**Figure 3:**
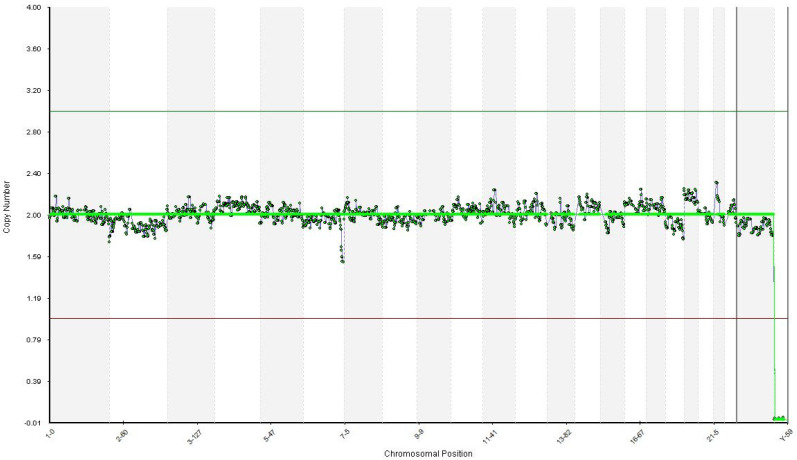
Preimplantation genetic screening results from NGS analysis of the embryo transferred. The x-axis indicates the chromosome numbers (1–22, X and Y) and the y-axis indicate the chromosome copy number assignments.

The amplification of the entire genome was performed using the SurePlex DNA Amplification System by Illumina Inc. 2018, California US. Using the BlueFuse Multi Analysis Software (Illumina Inc. 2018, San Diego, CA, USA), all 24 chromosomes were detected euploid for embryos. The identification of the mutation TTN gene on exon 358 (father’s mutation) and exon 260 (mother’s mutation) was performed only for euploid embryos using Sanger sequencing with specific primers on ABI 3500. PCR products for both embryos were purified and sequenced in both senses with a BigDye Terminator v3.1 Cycle Sequencing Kit by Thermo Fisher Scientific. Specific primers were manually designed according to both mutations and tested afterwards using blood samples from the parents. Both embryos tested by PGT-A were euploid. One of them was a carrier of the mother’s mutation c.48681C>G p.(Tyr16227), exon 260, and the other was a wild type (WT) for both mutations (Figure 3).

We performed a frozen-thawed embryo transfer in the following cycle, transferring the WT euploid embryo after endometrial preparation with exogenous estrogen. The result was positive, and we confirmed the ongoing viable pregnancy via ultrasound 14 days after.

Throughout the pregnancy, we performed the non-invasive double marker test (low-risk result) and fetal DNA analysis using maternal blood (low-risk result) and an invasive amniocentesis at 17 weeks of gestation, indicating a normal genetic profile. To test whether or not the second fetus presents a genetic abnormality, we extracted the DNA directly from the amniotic fluid. Targeted sequencing was performed on both DNA strands of the relevant TTN region. The reference sequence is TTN: NM_001267550.2. To exclude maternal cell contamination (MCC), we analyzed 15 STR autosomal markers plus amelogenin using the PowerPlex 16HS multiplex kit (Promega, Madison, Wisconsin, USA). Moreover, all the non-invasive ultrasound scans showed a normal growth rate and organ development. The evolution of the pregnancy was uneventful, and at 38 weeks, we carried out the C-section delivery of a healthy female baby of 2990 g, receiving an APGAR score of 9.

## 3. Discussion

As presented throughout this manuscript, IVF using PGT is a reliable strategy that strengthens the chances of obtaining a pregnancy in which one or both partners are healthy carriers of a potentially lethal mutation to the offspring. It should be mentioned that this is the second case in which the father’s mutation has been reported [19]. Accordingly, by combining distinct protocols, we were able to reveal two novel variants in parents affected by a genetic disease: c.48681C>G p.(Tyr16227) and c.104509_104510del p.(Leu34837Glufs*12), respectively, within the TTN gene in our case. Additionally, we highlighted in this case report the crucial role of PGT-A during such interventions and should be imperatively recommended to patients regardless of the attempt to prevent any risk of a possible aneuploidy. In order to offer a conclusive overview, we found it suitable to present in Table 1 all phenotype-genotype findings.

Rees et al. [20] published an article this year in which they presented a combined methodology aiming to improve the diagnosis efficiency from four distinct perspectives (genetic, biophysical, pathological and clinical) of the TTN-related myopathy and the associated pathogenicity of missense variants. From thirty patients identified and diagnosed with TTN-related congenital myopathy, two presumably had truncating variants or either one truncating and one missense TTN variant or homozygous for one TTN missense variant. According to their findings, the presentation was usually at birth, affected patients being dependent on ventilation. The most prevalent clinical signs were scoliosis, weakness, and contractures without involving the muscles. Therefore, this approach proposed was reliable in showing that congenital myopathy is strongly correlated with the existence of a truncating background or in homozygosity. Furthermore, they hypothesized that TTN destabilizing missense mutations phenocopy truncating variants could be a key feature of recessive cases.

From 504 patients with a known diagnosis of muscular dystrophy, congenital myopathy, or other skeletal muscle illnesses, only nine of them were positive either for titinopathy (*n* = 5) or TTN disease-causing variants (*n* = 4). More precisely, from those nine patients with novel titinopathies, five (55.5%) were men (0–46 years; SD, onset—25 years; 15.8). From the remaining four, three were men and one woman, possibly having disease-causing TNN variants from which 50% (*n* = 2) suffered from a congenital myopathy, and 50% (*n* = 2) exhibited a slowly progressive distal myopathy. Considering the large size of the TTN, it is hard through Sanger sequencing of candidate genes or extensive investigation to offer a conclusive diagnostic [21].

Carmignac et al. [12] identified five patients from two distinct consanguineous families from Morocco and Sudan affected by EOMFC. All five individuals presented impaired motor development and muscle-related weakness, especially at the proximal and distal lower limb levels. While the Moroccan patients (*n* = 3) exhibited showed specific onset in infancy, there was a delay for the other two Sudanese patients since the onset in the Sudanese individuals was pronounced at birth—neonatal hypotonia. All of them died eventually, but with the mention that four survived until their teenage stage. They developed progressive dilated cardiomyopathy with rhythm insufficiency.

Analogous with the aforementioned, Chauveau and co-authors [17] described a study that enrolled 31 patients from 23 families: five patients from four families with homozygous or heterozygous compounds. The authors identified five novel M-line truncating mutations in 17% of the patients, the heterozygous parents being clinically healthy. Precisely, they identified four novel titinopathies, involving distinct phenotypic changes, including cardiac septal defects, left ventricular non-compaction, arthrogryposis multiplex congenita or Emery–Dreifuss muscular dystrophy. The subsequent in vitro experiments revealed the absence of a functional titin kinase domain that was associated with a severe antenatal phenotype. In each individual’s case, the neurological or the muscular component was first affected.

Preimplantation genetic testing using karyomapping has proven its usefulness in obtaining healthy embryos and subsequent successful pregnancies in which myotonic dystrophy type I (MDI) [22,23,24,25], Duchenne muscular dystrophy (DMD) [26,27], muscular atrophy [28] are avoided. This approach has also been applied effectively for averting the consequences of other genetic events such as reciprocal translocation [29] and dynamic mutation [30].

Therefore, genetic diagnosis provides substantial support for prenatal decision-making and management. The current literature provides relevant studies that aim at strengthening the crucial role exome sequencing (ES) for prenatal diagnosis has in identifying fetal structural anomalies detected through various techniques—(ultra)sonography and the interplay with molecular protocols [31,32,33,34,35]. Here we also refer to the percentages attributed to positive/negative results, impact, utility and decision-making, patient status, and healthcare provider experiences. A point to consider is a recently published document under the auspices of the ACMG [36].

## 4. Conclusions

Based on all aspects presented in this case report, it can be concluded in accordance with the existing literature that severe congenital myopathy with fatal cardiomyopathy is one of the most debilitating inherited diseases. Furthermore, molecular biology can be viewed as an integrative component within the current IVF protocols. In this context, we were able to identify two novel variants following the searches performed on other databases. In parallel with the current state of knowledge, we consider it suitable to report the outcome of an IVF using PGT due to the nature of this disorder and its rarity.

## Figures and Tables

**Table 1:** Phenotype-genotype features of the family members.

Family Member	Phenotype	Genotype
First newborn	At 33 weeks of pregnancy minor clubfoot altered fetal biophysical profile	Three areas associated with loss of heterozygosity on chromosomes 1(q25.1–q25.3) of 6115 kb, 5(p15.2–p15.1) of 2589 kb and 8(q11.21–q11.23) of 4830 kb, a duplication of 1104 kb on chromosome 10 in the position q11.22, and a duplication of 1193 kb on chromosome 16 in the position p11.2p11.1.
	At 36 weeks of pregnancy abnormal fetal heart rate abnormal fetal movement poor muscular tonus	
	At birth generalized congenital muscular atony right diaphragmatic hernia cerebral atrophy neonatal anemia bilateral varus equinus neonatal hypocalcemia prematurity and low birth weight ostium secundum atrial heart defect tricuspid valve dysplasia right diaphragmatic hernia moderate cerebral atrophy	
Mother	Healthy carrier	Chr2(GRCh37):g.179479653G>C—TTN variant c.48681C>G p.(Tyr16227*)—Exon 260
Father	Healthy carrier	Chr2(GRCh37):g.179396832_179396833del—TTN variant c.104509_104510del p.(Leu34837Glufs*12)—Exon 358
First euploid embryo (W3)	Healthy embryo	Carrier of the mother’s mutation
Second euploid embryo (W4)	Healthy embryo	Healthy embryo (Wild-type) for both mutations

## Data Availability

The datasets used and analyzed during the current study are available from the corresponding author on reasonable request.

## References

[1] Haravuori H., Vihola A., Straub V., Auranen M., Richard I., Marchand S., Voit T., Labeit S., Somer H., Peltonen L. (2001). Secondary calpain3 deficiency in 2q-linked muscular dystrophy. Neurology.

[2] Hackman P., Vihola A., Haravuori H., Marchand S., Sarparanta J., De Seze J., Labeit S., Witt C., Peltonen L., Richard I. (2002). Tibial muscular dystrophy is a titinopathy caused by mutations in TTN, the gene encoding the giant skeletal-muscle protein titin. Am. J. Hum. Genet..

[3] Bang M.-L., Centner T., Fornoff F., Geach A.J., Gotthardt M., McNabb M., Witt C.C., Dietmar L., Gregorio C.C., Granzier H. (2001). The Complete Gene Sequence of Titin, Expression of an Unusual ≈700-kDa Titin Isoform, and Its Interaction With Obscurin Identify a Novel Z-Line to I-Band Linking System. Circ. Res..

[4] Savarese M., Sarparanta J., Vihola A., Udd B., Hackman P. (2016). Increasing Role of Titin Mutations in Neuromuscular Disorders. J. Neuromuscul. Dis..

[5] Hackman P., Udd B., Bönnemann C.G., Ferreiro A., Udd B., Hackman P., Ferreiro A., Bonnemann C., Beggs A., Gautel M. (2017). 219th ENMC International Workshop Titinopathies International database of titin mutations and phenotypes, Heemskerk, The Netherlands, 29 April–1 May 2016. Neuromuscul. Disord..

[6] Herman D.S., Lam L., Taylor M.R.G., Wang L., Teekakirikul P., Christodoulou D., Conner L., DePalma S.R., McDonough B., Sparks E. (2012). Truncations of Titin Causing Dilated Cardiomyopathy. N. Engl. J. Med..

[7] Gerull B., Gramlich M., Atherton J., McNabb M., Trombitás K., Sasse-Klaassen S., Seidman J.G., Seidman C., Granzier H., Labeit S. (2002). Mutations of TTN, encoding the giant muscle filament titin, cause familial dilated cardiomyopathy. Nat. Genet..

[8] Pfeffer G., Elliott H.R., Griffin H., Barresi R., Miller J., Marsh J., Evilä A., Vihola A., Hackman P., Straub V. (2012). Titin mutation segregates with hereditary myopathy with early respiratory failure. Brain.

[9] Pfeffer G., Barresi R., Wilson I.J., Hardy S.A., Griffin H., Hudson J., Elliott H.R., Ramesh A.V., Radunovic A., Winer J.B. (2014). Titin founder mutation is a common cause of myofibrillar myopathy with early respiratory failure. J. Neurol. Neurosurg. Psychiatry.

[10] Palmio J., Evilä A., Chapon F., Tasca G., Xiang F., Brådvik B., Eymard B., Echaniz-Laguna A., Laporte J., Kärppä M. (2014). Hereditary myopathy with early respiratory failure: Occurrence in various populations. J. Neurol. Neurosurg. Psychiatry.

[11] De Cid R., Ben Yaou R., Roudaut C., Charton K., Baulande S., Leturcq F., Romero N.B., Malfatti E., Beuvin M., Vihola A. (2015). A new titinopathy: Childhood-juvenile onset Emery-Dreifuss-like phenotype without cardiomyopathy. Neurology.

[12] Carmignac V., Salih M.A.M., Quijano-Roy S., Marchand S., Al Rayess M.M., Mukhtar M.M., Urtizberea J.A., Labeit S., Guicheney P., Leturcq F. (2007). C-terminal titin deletions cause a novel early-onset myopathy with fatal cardiomyopathy. Ann. Neurol..

[13] Salih M.A.M., Al Rayess M., Cutshall S., Urtizberea J.A., Al-Turaiki M.H.S., Ozo C.O., Straub V., Akbar M., Abid M., Andeejani A. (1998). A Novel Form of Familial Congenital Muscular Dystrophy in Two Adolescents. Neuropediatrics.

[14] Subahi S.A. (2001). Distinguishing Cardiac Features of a Novel Form of Congenital Muscular Dystrophy (Salih CMD). Pediatr. Cardiol..

[15] Fukuzawa A., Lange S., Holt M., Vihola A., Carmignac V., Ferreiro A., Udd B., Gautel M. (2008). Interactions with titin and myomesin target obscurin and obscurin-like 1 to the M-band—Implications for hereditary myopathies. J. Cell Sci..

[16] Pernigo S., Fukuzawa A., Bertz M., Holt M., Rief M., Steiner R.A., Gautel M. (2010). Structural insight into M-band assembly and mechanics from the titin-obscurin-like-1 complex. Proc. Natl. Acad. Sci. USA.

[17] Chauveau C., Bonnemann C.G., Julien C., Kho A.L., Marks H., Talim B., Maury P., Arne-Bes M.C., Uro-Coste E., Alexandrovich A. (2014). Recessive TTN truncating mutations define novel forms of core myopathy with heart disease. Hum. Mol. Genet..

[18] Salih M., Hamad M., Savarese M., Alorainy I., Alkhalidi H., Alotaibi A., Alsagob M., Colak D., Udd B., Kaya N. (2019). Delineating the phenotypes of early onset myopathy due to novel titin gene mutations. J. Neurol. Sci..

[19] Oates E.C., Jones K.J., Donkervoort S., Charlton A., Brammah S., Smith J.E., Ware J.S., Yau K.S., Swanson L.C., Whiffin N. (2018). Congenital Titinopathy: Comprehensive characterization and pathogenic insights. Ann. Neurol..

[20] Rees M., Nikoopour R., Fukuzawa A., Kho A.L., Fernandez-Garcia M.A., Wraige E., Bodi I., Deshpande C., Özdemir Ö., Daimagüler H.-S. (2021). Making sense of missense variants in TTN-related congenital myopathies. Acta Neuropathol..

[21] Savarese M., Maggi L., Vihola A., Jonson P.H., Tasca G., Ruggiero L., Bello L., Magri F., Giugliano T., Torella A. (2018). Interpreting Genetic Variants in Titin in Patients With Muscle Disorders. JAMA Neurol..

[22] Wang C.-W., Liu Y.-L., Chen C.-H. (2019). Targeting myotonic dystrophy by preimplantation genetic diagnosis-karyomapping. Taiwan J. Obstet. Gynecol..

[23] Kalman L., Tarleton J., Hitch M., Hegde M., Hjelm N., Berry-Kravis E., Zhou L., Hilbert J.E., Luebbe E.A., Moxley R.T. (2013). Development of a Genomic DNA Reference Material Panel for Myotonic Dystrophy Type 1 (DM1) Genetic Testing. J. Mol. Diagn..

[24] Senba H., Sueoka K., Sato S., Higuchi N., Mizuguchi Y., Sato K., Tanaka M. (2020). The impact of parental unaffected allele combination on the diagnostic outcome in the preimplantation genetic testing for myotonic dystrophy type 1 in Japanese ancestry. Reprod. Med. Biol..

[25] Lian M., Lee C.G., Chong S.S. (2019). Robust Preimplantation Genetic Testing Strategy for Myotonic Dystrophy Type 1 by Bidirectional Triplet-Primed Polymerase Chain Reaction Combined with Multi-microsatellite Haplotyping Following Whole-Genome Amplification. Front. Genet..

[26] Mongkolchaipak S., Piyamongkol W., Piyamongkolx W. (2019). 62. Successful Strategy of Comprehensive Pre-Implantation Genetic Testing for Duchenne Muscular Dystrophy and Chromosome Balance Using Karyomapping. Reprod. Biomed. Online.

[27] Bianco B., Christofolini D.M., Conceição G.S., Barbosa C.P. (2017). Preimplantation genetic diagnosis associated to Duchenne muscular dystrophy. Einstein.

[28] Fu Y., Shen X., Wu H., Chen D., Zhou C. (2019). Preimplantation Genetic Testing for Monogenic Disease of Spinal Muscular Atrophy by Multiple Displacement Amplification: 11 unaffected livebirths. Int. J. Med. Sci..

[29] Beyer C.E., Lewis A., Willats E., Mullen J. (2019). Preimplantation genetic testing using Karyomapping for a paternally inherited reciprocal translocation: A case study. J. Assist. Reprod. Genet..

[30] Shi D., Xu J., Niu W., Liu Y., Shi H., Yao G., Shi S., Li G., Song W., Jin H. (2020). Live births following preimplantation genetic testing for dynamic mutation diseases by karyomapping: A report of three cases. J. Assist. Reprod. Genet..

[31] Pratt M., Garritty C., Thuku M., Esmaeilisaraji L., Hamel C., Hartley T., Millar K., Skidmore B., Dougan S., Armour C.M. (2020). Application of exome sequencing for prenatal diagnosis: A rapid scoping review. Genet. Med..

[32] Dempsey E., Haworth A., Ive L., Dubis R., Savage H., Serra E., Kenny J., Elmslie F., Greco E., Thilaganathan B. (2021). A report on the impact of rapid prenatal exome sequencing on the clinical management of 52 ongoing pregnancies: A retrospective review. BJOG Int. J. Obstet. Gynaecol..

[33] Lord J., McMullan D.J., Eberhardt R.Y., Rinck G., Hamilton S.J., Quinlan-Jones E., Prigmore E., Keelagher R., Best S.K., Carey G.K. (2019). Prenatal exome sequencing analysis in fetal structural anomalies detected by ultrasonography (PAGE): A cohort study. Lancet.

[34] Chen M., Chen J., Wang C., Chen F., Xie Y., Li Y., Li N., Wang J., Zhang V.W., Chen D. (2020). Clinical application of medical exome sequencing for prenatal diagnosis of fetal structural anomalies. Eur. J. Obstet. Gynecol. Reprod. Biol..

[35] Drury S., Williams H., Trump N., Boustred C., Lench N., Scott R.H., Chitty L.S., GOSGene (2015). Exome sequencing for prenatal diagnosis of fetuses with sonographic abnormalities. Prenat. Diagn..

[36] Monaghan K.G., Leach N.T., Pekarek D., Prasad P., Rose N.C. (2020). on behalf of the ACMG Professional Practice and Guidelines Committee. The use of fetal exome sequencing in prenatal diagnosis: A points to consider document of the American College of Medical Genetics and Genomics (ACMG). Genet. Med..

